# The Protective Role of the Ratio of Arterial Partial Pressure of Oxygen and Fraction of Inspired Oxygen after Re-Supination in the Survival of Patients with Severe COVID-19 Pneumonia

**DOI:** 10.2174/0118743064334878241028114347

**Published:** 2024-11-14

**Authors:** Jesús S. Sánchez-Díaz, Karla G. Peniche-Moguel, Diego Escarramán-Martínez, José M. Reyes-Ruíz, Orlando R. Pérez-Nieto

**Affiliations:** 1 Instituto Mexicano del Seguro Social, Terapia Intensiva, Mexico; 2 Secretaria de Salud, Terapia Intensiva, Mexico

**Keywords:** Prone position, Supine position, Pneumonia, Respiratory distress syndrome, PaO_2_/FiO_2_ ratio, Protective factors

## Abstract

**Background:**

The role of the ratio between the arterial partial pressure of oxygen and the inspired fraction of oxygen (PaO_2_/FiO_2_ ratio) during the change in position is not fully established.

**Methods:**

This retrospective, single-center cohort study included 98 patients with severe COVID-19 pneumonia.

**Objective:**

This study aimed to evaluate the predictive value of the PaO_2_/FiO_2_ ratio for survival in patients with severe COVID-19 pneumonia between changing from supine to prone positions and *vice versa*. The PaO_2_/FiO_2_ ratio was measured preproning (T0), 30 min to 1 hour (T1), and 48 h after prone positioning (T2), and 30 min to 1 h after re-supination (T3).

**Results:**

The PaO_2_/FiO_2_ ratio at T2 and T3 was higher in the survivors than in the non-survivors (T2= 251.5 *vs*. 208.5, *p*= 0.032; T3= 182 *vs*. 108.5, *p*<0.001). The PaO_2_/FiO_2_ ratio at T3 was an independent protective factor (Hazard Ratio (HR)= 0.993; 95% Confidence Interval (CI)= 0.989-0.998; *p*= 0.006) for survival. A threshold of ≤129 for the PaO_2_/FiO_2_ ratio at T3 predicted non-survival with a sensitivity and specificity of 67.86 and 80.95, respectively (Area Under the Curve (AUC)= 0.782; 95% CI 0.687-0.859).

**Conclusion:**

The PaO_2_/FiO_2_ ratio is a significant protective factor of survival in severe COVID-19 pneumonia within 30 min-1 hour after returning to the supine position (re-supination).

## 1 INTRODUCTION

The benefits of the prone position in patients with acute respiratory failure have been documented since 1976 [1]. Improved gas exchange with an increased arterial oxygen partial pressure (PaO_2_) and a decreased arterial partial pressure of carbon dioxide (PaCO_2_) in Acute Respiratory Distress Syndrome (ARDS) is well described [2]. However, the most important thing regarding the prone position is the decrease in mortality when it is implemented early (< 48 hours); it is especially used once ARDS has been diagnosed, and for at least 16 hours in patients with severe hypoxemia (PaO_2_/FiO_2_ <150 mmHg) [3-5]. The pneumonia caused by Severe Acute Respiratory Syndrome Coronavirus 2 (SARS-CoV-2) has been identified as Coronavirus Disease 2019 (COVID-19). Up to 80% of COVID-19-related intubated patients have died [6, 7]. Therefore, guidelines for the care of critically ill adult patients with COVID-19 have recommended the use of the prone position [8]. The use of protective mechanical ventilation in terms of airway volume and pressure, including the prone position, is another strategy that can reduce the fatal outcome at 28 days (RR 0.74, 95% CI 0.61-0.88) [9]. Thus, the prone position should be accompanied by a low Tidal Volume (VT) [10], limiting plateau Pressure (Pplat) [11], Mechanical Power (MP) [12], Driving Pressure (DP) [13], and favorable Ventilatory Efficiency (VE) [14]. Together, these interventions could reduce the risk of Ventilator-induced Lung Injury (VILI), which is a reflection of inappropriate pressures and volumes, *i.e*., excessive stress and strain [15].

Invasive Mechanical Ventilation (IMV) in the supine position does not fully benefit lung function; the different gravitational forces between the dependent and nondependent regions cause the pleural Pressure (Ppl) to be more negative in non-dependent areas, which increases transpulmonary Pressure (Ptpl) and causes greater alveolar distension. In dependent areas, where Ppl is less negative, the opposite effect is obtained, and as a result, Ptpl is lower, translating into less alveolar distension [16]. Ventilation in the prone position favors the homogeneous distribution of Ptpl that generates more uniform ventilation, improves the diaphragmatic excursion of the dorsal region, and decreases the “mechanical effect” of the mediastinal structures (mainly the heart), redistributing ventilation. On the other hand, ventilation in the prone position causes redistribution of pulmonary blood flow, improving the ventilation/perfusion (V/Q) ratio [17]. Finally, respiratory mechanics benefit from improved distensibility of the chest wall and lung parenchyma. Together, these changes increase the PaO_2_/FiO_2_ ratio and decrease PaCO_2_ [2, 18]. It has been proposed that a “prolonged” (more than 36 hours) prone position is safe and free of major adverse events in patients with ARDS secondary to SARS-CoV-2; likewise, the improvement could be greater and sustained [19]. However, in many patients, the improvement in the PaO_2_/FiO_2_ ratio is not sustained after resupination, which could be associated with patient outcomes. Therefore, our research aimed to determine survival by observing the change in the values of this index from the supine position to the prone position and *vice versa*.

## 2 MATERIAL AND METHODS

### 2.1 Study Population

This was a longitudinal retrospective observational cohort study. The data were collected from October 2, 2023, to November 30, 2023. The data of the patients who met the inclusion criteria and were admitted to the Intensive Care Unit (ICU) of a tertiary hospital were recorded. The data consisted of the medical records of patients who had met the inclusion criteria. The family members of the research participants signed the written informed consent for admission to intensive care and for the use of medical data for research purposes without the publication of their name or social security number that could contribute to their identification. Patients over 18 years of age were included and children were not included.

### 2.2 Patients

Convenience sampling was performed, and the records of patients admitted to the ICU with a diagnosis of severe COVID-19 pneumonia were included. The inclusion criteria were age ≥18 years, SARS-CoV-2 infection confirmed by Reverse Transcriptase-Polymerase Chain Reaction (RT‒PCR) test, ARDS defined according to the Berlin criteria [20], having received invasive mechanical ventilation, and prone position [21] for at least 48 continuous hours, as part of the treatment. Patients in the prone position for less than 48 continuous hours and patients with incomplete medical records were excluded. The prone position was considered for those patients with a ratio between partial pressure of oxygen and inspired fraction of oxygen (PaO_2_/FiO_2_) <150 mmHg, Positive End Expiratory Pressure (PEEP) ≥5 cmH_2_O, and FiO_2_ ≥0.6. Ventilatory support was carried out with Puritan Bennett 840 ventilators. This was a non-intervention study so the informed consent present in the medical records was that of admission to the ICU.

### 2.3 Data Collection

Data of the patients meeting the inclusion criteria were obtained from the electronic medical records. Clinical parameters, including sex, age, comorbidities, and respiratory parameters, were collected on ICU admission.

### 2.4 Definitions

Prone and supine positioning and monitoring of respiratory parameters. The prolonged prone position was defined as the anatomical position of the human body lying face down and with the head on one side, the neck in a neutral position, and the thoracic extremities extended and placed next to the trunk for more than 48 continuous hours [22]. After orotracheal intubation of patients with severe COVID-19 pneumonia, the PaO_2_/FiO_2_ ratio was measured pre-proning (T0), 30 min to 1 hour (T1), and 48 h after prone positioning (T2), and 30 min to 1 h after re-supination (T3). The PaO_2_/FiO_2_ ratio was obtained as the ratio between the partial pressure of oxygen (PaO_2_) and the fraction of inspired oxygen (FiO_2_). The Driving Pressure of the respiratory system (DP) was calculated as the difference between Plateau Pressure (Pplat) and the levels of Positive End Expiratory Pressure (PEEP). Compliance of the Respiratory System (CRS) was defined as the product between Tidal Volume (VT) and DP. Mechanical Power (MP) was calculated according to Gattinoni´s simplified formula: 0.098 x Respiratory Rate (RR) x VT x (peak Pressure (Ppeak) – (PPLAT - PEEP/2)). Ventilatory Ratio (VR) was determined as (minute ventilation (ml/min) x PaCO_2_ (mmHg))/(predicted body weight (kg) x 100 x 37.5).

### 2.5 Statistical Analysis

Data have been reported as numbers (percentage) for categorical variables and mean (Standard Deviation (SD)) or median (interquartile range (IQR)) for continuous variables. The Shapiro‒Wilk test was used to evaluate the normality assumption of continuous variables. Non- normally distributed continuous variables were compared using the Mann‒Whitney U-test, and normally distributed continuous variables were compared with the Student’s t-test. The categorical variables between the groups were compared using the chi-square test or Fisher´s exact test. Friedman´s test was used to compare the PaO_2_/FiO_2_ ratio at T0, T1, T2, and T3 within each group, and if statistical significance was detected, multiple comparisons were carried out with Wilcoxon´s signed-rank test for post-hoc comparisons between the related values. The bivariate correlation between the variables under study was assessed using the Spearman correlation coefficient. A univariate Cox regression analysis was performed, and only the variables significantly associated with the non-survival of COVID-19 patients were entered into a multivariate model with a stepwise method. In this regard, the Hazard Ratios (HRs) together with 95% Confidence Intervals (CIs) were estimated. Receiver Operating Characteristic (ROC) curve analysis was applied to evaluate the predictive value of the PaO_2_/FiO_2_ ratio, considering the Area Under the Curve (AUC). The Kaplan-Meir survival curve to estimate survival according to the best cutoff point of the PaO_2_/FiO_2_ ratio at T3 was constructed. The statistical significance of the differences between the categories of PaO_2_/FiO_2_ ratio was calculated using the log-rank test. A p-value <0.05 was considered statistically significant. Statistical analysis was performed using R Studio (version 1.0.153), SPSS 25.0 (SPSS Inc., Chicago, IL, USA), and MedCalc (Software 8.1.1.0; Mariakerke, Belgium).

## 3 RESULTS

**Fig. (1):**
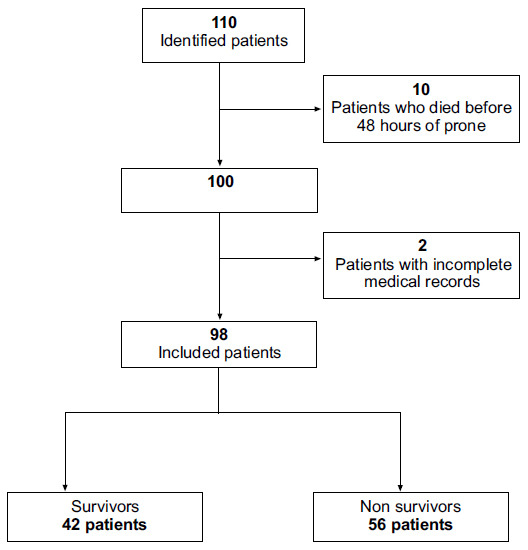
Flowchart.

98 patients with a diagnosis of severe COVID-19 pneumonia were included in the statistical analysis; they were classified as survivors and non-survivors (Fig. **1**). Demographic and clinical characteristics by subgroup are described in Table **1**. The survivor group had a median age of 60.4 years old ±14.75 compared to 66 years old ±11.09 in the non-survivor group; the difference exhibited statistical significance.

Among the variables that were considered of interest for the clinical evolution of patients with severe COVID-19 pneumonia, sex, age, medical comorbidities, Pressure plateau (Pplat), Driving Pressure (ΔP), Compliance of the Respiratory System (CrS), Mechanical Power (MP), and Ventilatory Ratio (VR) stood out (Table **1**). In all patients, the following variables were calculated: the PaO_2_/FiO_2_ ratio was measured pre-proning (T0), 30 min to 1 hour (T1), and 48 h after prone positioning (T2), and 30 min to 1 h after resupination (T3). The findings have emphasized a PaO2/FIO2 value in the highest T3 in the survivors group compared to non-survivors, *i.e*., 182mmHg (105.5mmHg) and 108.5mmHg (68mmHg), respectively (p=0.001; Table **1**).

The univariate and multivariate analyses of the variables of interest have highlighted age (>55 years old) with HR 1.045 (95% CI 1.016-1.074; p=0.001) to lead to fatal outcomes. Likewise, T3 (after re-supination (after 48 hours in prone position)) has exhibited an HR of 0.993 (95% CI 0.989-0.998; p=0.006; Table **2**).

**Fig. (2):**
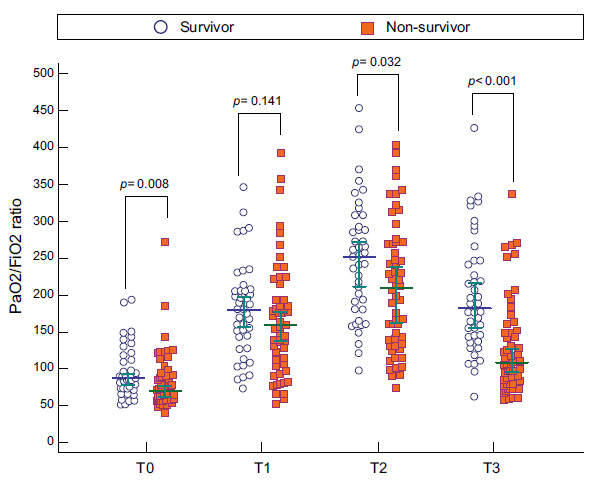
Distribution of PaO_2_/FiO_2_ ratio levels categorized into survival and non-survival groups during the intensive care unit stay. A Wilcoxon rank sum test was performed. Statistical significance was determined at *p* < 0.05. T0= values of the PaO_2_/FiO_2_ ratio in the supine position after intubation; T1= values of the PaO_2_/FiO_2_ ratio during the session 30 min to 1 hour after the patient was placed in the prone position; T2= values of the PaO_2_/FiO_2_ ratio 48 h after prone positioning; T3= values of the PaO_2_/FiO_2_ ratio between 30 min to 1 h after the patient had returned to the supine position.

**Fig. (3):**
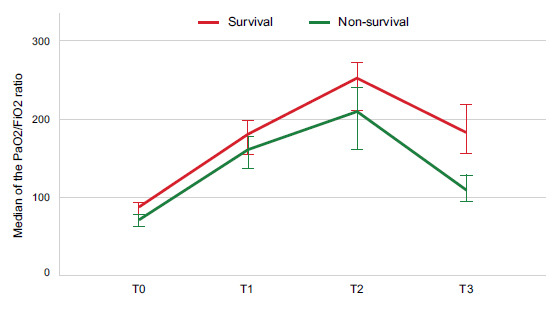
Dynamic changes in the PaO_2_/FiO_2_ ratio in patients with COVID-19 in the ICU. PaO_2_/FiO_2_ ratio level curve of different measurements during T0, T1, T2, and T3 between the survival and non-survival groups. Friedman´s test was used to study the changes over time within each group. Statistical significance was determined at *p* < 0.05. T0= values of the PaO_2_/FiO_2_ ratio in the supine position after intubation; T1= values of the PaO_2_/FiO_2_ ratio during the session 30 min to 1 hour after the patient was placed in the prone position; T2= values of the PaO_2_/FiO_2_ ratio 48 h after prone positioning; T3= values of the PaO_2_/FiO_2_ ratio between 30 min to 1 h after the patient had returned to the supine position.

**Fig. (4):**
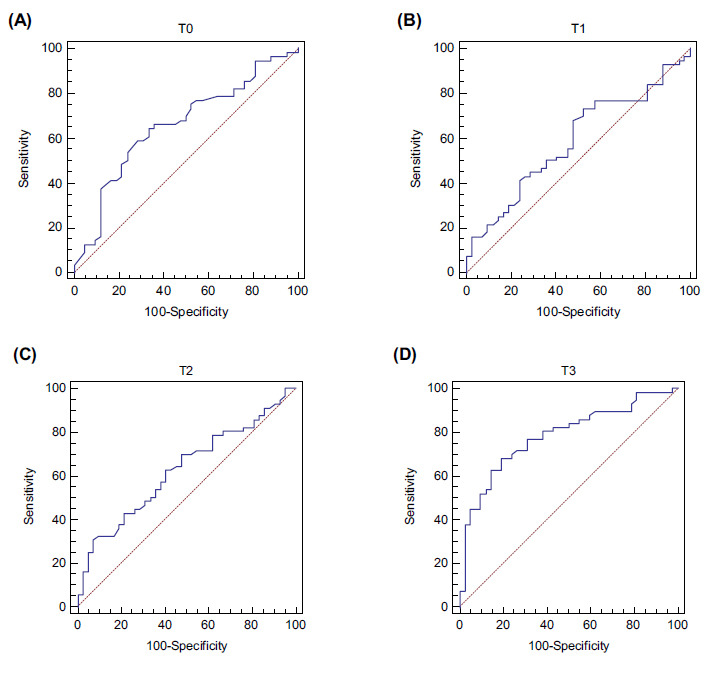
ROC curve model of T0 (**A**), T1 (**B**), T2 (**C**), and T3 (**D**) for prediction of non-survival in severe COVID-19 pneumonia. T0= values of the PaO_2_/FiO_2_ ratio in the supine position after intubation; T1= values of the PaO_2_/FiO_2_ ratio during the session 30 min to 1 hour after the patient was placed in the prone position; T2= values of the PaO_2_/FiO_2_ ratio 48 h after prone positioning; T3= values of the PaO_2_/FiO_2_ ratio between 30 min to 1 h after the patient had returned to the supine position.

When comparing the PaO_2_/FiO_2_ in the groups of survivors and non-survivors, T2 stood out as 251.5mmHg and 208.5mmHg, respectively (p= 0.032), and T3 as 182mmHg and 108.5mmHg, respectively (*p* <0.001; Fig. **2**). The dynamic changes in the PaO_2_/FiO_2_ ratio at T0, T1, T2, and T3 within each group were also evaluated in this study. With the Friedman test, it was highlighted that the group of survivors presented high levels of PaO_2_/FiO_2_ for time T0, T1, T2, and T3 compared to the group of non-survivors (T0 = 87 (45.25), T1 = 180 (66.5), T2= 251.5 (115.25), T3= 182 (105.5), consecutively (*p* <0.00001 in all cases)) (Fig. **3**). On the other hand, the PaO_2_/FiO_2_ ratio was increased at T2 compared to T0, T1, and T3 in the non-survivors )T0= 70.5 (30.75) *vs*. T1= 160 (85.5) *vs*. T2= 208.5 (136.25) *vs*. T3= 108.5 (68), *p* <0.00001). Through the ROC curve analysis, the ratio was compared at different times **(**Fig. **4A**-**D****).** Additionally, the results of the Area Under the Curve (AUC), best cutoff point, Youden´s index, Sensitivity (S), Specificity (Sp), Positive and Negative Predictive Values (PPV, NPV), and likelihood ratios for the PaO_2_/FiO_2_ ratio at T0, T1, T2, and T3 are shown in Table **3**.

**Fig. (5):**
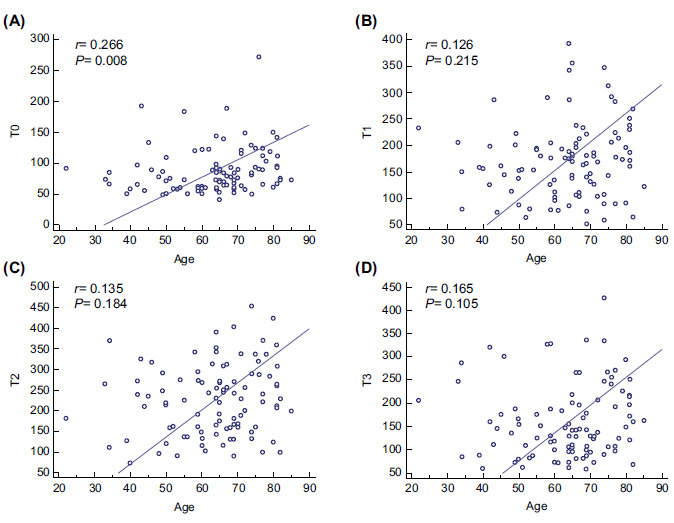
Bivariate correlations of age with the PaO_2_/FiO_2_ ratio at T0, T1, T2, and T3. In all severe COVID-19 pneumonia categories, age was positively correlated with T0 (**A**), but this variable was not correlated with T1 (**B**), T2 (**C**), and T3 (**D**). Spearman´s test was used to evaluate the correlation. Statistical significance was determined at *p* < 0.05. Definition of abbreviations: T0= values of the PaO_2_/FiO_2_ ratio in the supine position after intubation; T1= values of the PaO_2_/FiO_2_ ratio during the session 30 min to 1 hour after the patient was placed in the prone position; T2= values of the PaO_2_/FiO_2_ ratio 48 h after prone positioning; T3= values of the PaO_2_/FiO_2_ ratio between 30 min to 1 h after the patient had returned to the supine position.

**Fig. (6):**
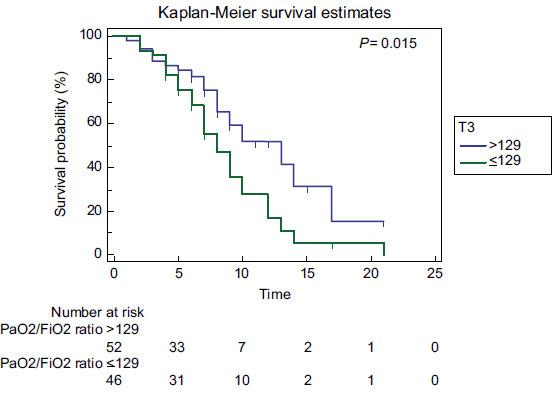
Kaplan‒Meier survival curve according to the PaO_2_/FiO_2_ ratio level groups: high levels (>129; blue line) *versus* low levels (≤129; green line). T3= values of the PaO_2_/FiO_2_ ratio between 30 min to 1 h after the patient had returned to the supine position. Statistical significance was determined at *p* < 0.05.

The PaO_2_/FiO_2_ ratio at time T3 of <129mmHg presented an AUC of 0.782 with S and Sp of 67.8% and 80.9%, respectively (Table **3**). The PaO_2_/FiO_2_ ratio at T0 was positively correlated with age (r= 0.266, p= 0.008), whereas the PaO_2_/FiO_2_ ratio at T1 (r= 0.126, p= 0.215), T2 (r= 0.135, p= 0.184), and T3 (r= 0.165, p= 0.105) showed no significant correlation with age (Fig. **5A**-**D**). A Kaplan‒Meier survival curve was plotted (Fig. **6**) according to the best cutoff point (≤129mmHg) of the PaO_2_/FiO_2_ ratio at T3, which demonstrated a lower survival over time (p= 0.015).

## 4 DISCUSSION

In this study, our goal was to avoid both hypoxemia and hyperoxemia. PaO_2_ levels between 60-100mmHg [23] and peripheral oxygen saturation (SpO_2_) between 92% and 96% indicate COVID-19 infection in patients [24]. An SpO_2_ of < 92% has been reported to indicate an increased risk of mortality (HR 1.62; 95% CI 1.02-2.56) [25] as well as an SpO_2_ > 96% (RR 1.21; 95% CI 1.03-1.43) [26]. When possible, besides SpO_2_, the sigmoid shape of the oxygen dissociation curve should be considered. Pulse oximetry between 92% and 96% can represent a PaO_2_ between 60 and 200 mmHg, which can be an extremely different value with an important connotation regarding the treatment [27].

Variations in PaO_2_/FiO_2_ when placing the patient in the prone position or at weaning are important, but the benefit of this position goes beyond a variable. It has been documented that there is a 53% increase in PaO_2_/FiO_2_ in patients with moderate to severe ARDS; 12 hours after its start, the prone position improves survival HR 0.11 (95% CI 0.05-0.25, p=<0.001) [28]. In contrast, the decrease in the PaO_2_/FiO_2_ value upon returning to the supine position is associated with an increase in the need for tracheostomy and even mortality. In addition, the “sustained improvement” of PaO_2_/FiO_2_ when returning to the supine position is independently associated with extubation success (RR 1.563, 95% CI 1.329-1.838, p = <0.001) [29]. Similar to this, our research has demonstrated higher PaO_2_/FiO_2_ values at both T2 and T3 in the survivor group. However, only the PaO_2_/FiO_2_ ratio at T3 (HR= 0.993; 95% CI 0.989-0.998, p= 0.006) has been an independent predictor of death in COVID-19 patients with ARDS. The survivor group had a higher level of CRS (35.92 ± 9.15 *vs*. 30.37 ±9.38, *p* <0.005) than the non-survival group. This variable has been reported to be associated with discharge from the ICU at 28 days, but to not be a predictor of mortality [30].

The elimination of CO_2_ is as important as the increase in oxygenation. Recruitment of well-perfused but poorly ventilated units decreases shunts and, thus, favors CO_2_ elimination. The Ventilatory Ratio (VR) is a simple parameter that assesses alveolar ventilation. Its normal value is ~1, without units. The VR is mainly determined by dead-space fraction (VD/VT) and is an independent predictor of mortality. Among patients with ARDS, VR has been found to be higher in non-survivors [31]. Among patients with COVID-19 who required invasive mechanical ventilation, the increase in VR from ICU admission compared to day 3 was associated with mortality (OR 1.4, CI 1.01-1.07, p= 0.030), regardless of PaO_2_/FiO_2_ variations (OR 0.99, CI 0.95-1.02, p= 0.47) [32]. Dead space could predict mortality in patients with ARDS [33]; however, in our patient population, there were no differences in VR between the survivors and non-survivors or in PPLAT, DP, and MP between the survivors and non-survivors. Thus, it can be stated that a prolonged prone position (~48 hours) is viable, and at a greater number of hours, the benefits are greater, and there is no increase in the inherent risks of this anatomical position. Even a prolonged prone position could be useful for preserving oxygenation improvement after resupination [34-36].

The limitations of our study are stated as follows: the study was carried out at a single center, it was retrospective, the sample size was small, the mechanical ventilation protocol was not standardized (although the protective ventilation strategy was used), and we did not report adverse events of any kind regarding the prolonged prone position. However, our strength is that we have reported patients to share homogeneous baseline characteristics.

## CONCLUSION

The results obtained in this study, although analyzed retrospectively, have suggested the dynamic evolution of lung disease in severe COVID-19 pneumonia, with age being an independent variable for fatal outcomes; however, what was most notable is the evolution of the ratio PaO_2_/FiO_2_ at different times in the clinical evolution, that is, the PaO_2_/FiO_2_ values measured in T2 could translate the gasometric paremeters’ response to the orotracheal intubation maneuver and prone position, but the T3 time (re-supination) was the most predictive for the final outcome.

## Figures and Tables

**Table 1:** Demographics and clinical characteristics of patients with severe COVID-19 pneumonia.

**Variable**	**Total (n= 98)**	**Survivors (n = 42)**	**Non-survivors (n = 56)**	***p*-value**
Female, n (%)	34 (34.7)	13 (31)	21 (37.5)	0.50^a^
Age (years)	63.6 ±13.01	60.4 ±14.75	66 ±11.09	**0.035^b^**
BMI (kg m^-2^)	32.66 (6.72)	32.65 (6.67)	33.67 (7.55)	0.135^c^
Diabetes, n (%)	45 (45.9)	13 (31)	32 (57.1)	**0.01^a^**
AH, n (%)	65 (66.3)	23 (54.8)	42 (75)	**0.036^a^**
Smoking, n (%)	32 (32.7)	8 (19)	24 (42.9)	**0.013^a^**
CKD, n (%)	7 (7.1)	1 (2.4)	6 (10.7)	0.233^d^
Cardiopathy, n (%)	6 (6.1)	4 (9.5)	2 (3.6)	0.397^d^
Lactate (mg/dL)	1.6 (0.9)	1.7 (0.85)	1.6 (0.78)	0.297^b^
P_PLAT_ (cmH_2_O)	23.37 ±3.47	22.90 ±3.27	23.73 ±3.60	0.245^b^
ΔP (cmH_2_O)	14.62 ±3.25	14.47 ±3.27	14.73 ±3.26	0.702^b^
C_RS_ (ml/cmH_2_O)	32.75 ±9.64	35.92 ±9.15	30.37 ±9.38	**0.004^b^**
MP (J/min)	18.59 (7.97)	18.45 (7.31)	18.81 (8.81)	0.983^c^
VR	1.84 (0.72)	1.88 (0.73)	1.82 (0.70)	0.994^c^
T0	76.5 (36.5)	87 (41.25)	70.5 (30.75)	**0.008^c^**
T1	171 (84.5)	180 (66.5)	160 (85.5)	0.141^c^
T2	226.5 (126)	251.5 (115.25)	208.5 (136.25)	**0.032^c^**
T3	141 (103.75)	182 (105.5)	108.5 (68)	**<0.001^c^**

**Note: **Data are presented as mean [± Standard Deviation (SD)], median [Interquartile Range (IQR)], or number (percentage). *P* values were calculated using a = chi-square test; b= Student *t*-test; c= Mann-Whitney U test; d= Fisher´s exact test. Statistical significance was determined at *p*< 0.05.

Definition of abbreviations: BMI= body mass index; AH= arterial hypertension; CKD= chronic kidney disease; P_PLAT_= plateau pressure; ΔP= driving pressure of respiratory system; C_RS_= compliance of the respiratory system; MP= mechanical power; VR= ventilatory ratio; T0= value of the PaO_2_/FiO_2_ ratio in the supine position after intubation; T1= value of the PaO_2_/FiO_2_ ratio during the session 30 min to 1 hour after the patient was placed into prone position; T2= value of the PaO_2_/FiO_2_ ratio between 1 h to 8 h after prone positioning; T3= value of the PaO_2_/FiO_2_ ratio between 30 min to 1 h after the patient had returned to the supine position.

**Table 2:** Univariable and multivariable COX regression analyses of mortality in patients with severe COVID-19 pneumonia.

**Variable**	**Univariable**			**Multivariable**		
	**HR**	**(95% CI)**	**P-value**	**HR**	**(95% CI)**	**P-value**
Female	1.4	0.815-2.424	0.223	-	-	-
Age	1.03	1-1.05	**0.012**	1.045	1.016-1.074	**0.001**
BMI	1.02	0.993-1.06	0.116	-	-	-
Diabetes	1.37	0.8-2.33	0.244	-	-	-
AH	1.59	0.871-2.918	0.131	-	-	-
Smoking	1.38	0.817-2.363	0.061	-	-	-
CKD	1	0.422-2.378	0.996	-	-	-
Cardiopathy	0.82	0.20-3.39	0.794	-	-	-
Lactate	0.91	0.6-1.37	0.659	-	-	-
P_PLAT_	1.03	0.95-1.11	0.389	-	-	-
ΔP	1.03	0.95-1.12	0.432	-	-	-
C_RS_	0.98	0.95-1.01	0.22	-	-	-
MP	0.996	0.953-1.041	0.873	-	-	-
VR	1.122	0.738-1.704	0.591	-	-	-
T0	0.997	0.989-1	0.514	-	-	-
T1	0.998	0.995-1	0.542	-	-	-
T2	0.997	0.993-1	0.073	-	-	-
T3	0.9958	0.9917-0.9998	**0.042**	0.993	0.989-0.998	**0.006**

**Note: **Candidate predictors with statistically significant differences (*p*< 0.05) in univariate Cox analysis and multivariable Cox regression analysis were included using a step-wise method. Hazard Ratio (HR) and 95% Confidence Interval (95% CI) have been reported. Statistically significant *p* values (<0.05) have been highlighted in bold. Statistical significance was determined at *p* < 0.05.

Definition of abbreviations: BMI= body mass index; AH= arterial hypertension; CKD= chronic kidney disease; P_PLAT_= plateau pressure; ΔP= driving pressure of respiratory system; C_RS_= compliance of the respiratory system; MP= mechanical power; VR= ventilatory ratio; T0= value of the PaO_2_/FiO_2_ ratio in the supine position after intubation; T1= value of the PaO_2_/FiO_2_ ratio during the session 30 min to 1 hour after the patient was placed in prone position; T2= value of the PaO_2_/FiO_2_ ratio between 1 h to 8 h after prone positioning; T3= value of the PaO_2_/FiO_2_ ratio between 30 min to 1 h after the patient had returned to the supine position.

**Table 3:** Best cut-off, Youden’s index, sensitivity, specificity, negative and positive predictive values, and positive and negative likelihood ratio of the value of PaO_2_/FiO_2_ ratio for mortality due to severe COVID-19 pneumonia.

**Variable**	**AUC**	**Best Cut-off**	**Youden’s Index**	**Sensitivity (%)**	**Specificity (%)**	**p-value**	**NPV**	**PPV**	**-LR**	**+LR**
T0	0.656	≤77	0.309	64.29	66.67	0.005	0.58	0.72	0.54	1.93
T1	0.587	≤185	0.208	73.21	47.62	0.133	0.57	0.65	0.56	1.40
T2	0.627	≤142	0.232	30.36	92.86	0.024	0.50	0.85	0.75	4.25
T3	0.782	≤129	0.488	67.86	80.95	<0.0001	0.65	0.82	0.40	3.56

**Note: **Definition of abbreviations: T0= value of the PaO_2_/FiO_2_ ratio in the supine position after intubation; T1= value of the PaO_2_/FiO_2_ ratio during the session 30 min to 1 hour after the patient was placed in prone position; T2= value of the PaO_2_/FiO_2_ ratio between 1 h to 8 h after prone positioning; T3= value of the PaO_2_/FiO_2_ ratio between 30 min to 1 h after the patient had returned to the supine position; AUC, area under the curve; NPV, negative predictive value; PPV, positive predictive value; -LR, negative likelihood ratio; +LR, positive likelihood ratio.

## Data Availability

This study is available at: https://zenodo.org/me/ requests/1cab2455-7ad0-4867-a12a-a3903fe8b9ae.

## LIST OF ABBREVIATIONS

PaO_2_/FiO_2_: = Arterial partial pressure of oxygen and inspired fraction of oxygen
ARDS: = Acute respiratory distress syndrome
COVID-19: = Coronavirus disease 2019
SARS-CoV-2: = Severe acute respiratory syndrome coronavirus 2

## STANDARDS OF REPORTING

The research was conducted based on the Strengthening the Reporting of Observational Studies in Epidemiology (STROBE) methodology for observational studies.
